# Association between Body Temperature Patterns and Neurological Outcomes after Extracorporeal Cardiopulmonary Resuscitation

**DOI:** 10.1371/journal.pone.0170711

**Published:** 2017-01-23

**Authors:** Jeong-Am Ryu, Taek Kyu Park, Chi Ryang Chung, Yang Hyun Cho, Kiick Sung, Gee Young Suh, Tae Rim Lee, Min Seob Sim, Jeong Hoon Yang

**Affiliations:** 1 Department of Critical Care Medicine, Samsung Medical Center, Sungkyunkwan University School of Medicine, Seoul, Republic of Korea; 2 Division of Cardiology, Department of Medicine, Samsung Medical Center, Sungkyunkwan University School of Medicine, Seoul, Republic of Korea; 3 Department of Thoracic and Cardiovascular Surgery, Samsung Medical Center, Sungkyunkwan University School of Medicine, Seoul, Republic of Korea; 4 Division of Pulmonary and Critical Care Medicine, Department of Medicine, Samsung Medical Center, Sungkyunkwan University School of Medicine, Seoul, Korea; 5 Department of Emergency Medicine, Samsung Medical Center, Sungkyunkwan University School of Medicine, Seoul, Republic of Korea; Azienda Ospedaliero Universitaria Careggi, ITALY

## Abstract

We evaluated the association of body temperature patterns with neurological outcomes after extracorporeal cardiopulmonary resuscitation (ECPR). Between December 2013 and December 2015, we enrolled 48 patients with cardiac arrest who survived for at least 24 hours after ECPR. Based on their body temperature patterns and the intention to control fever, we divided the patients into those in whom fever was actively controlled (*N* = 25), those with normothermia (*N* = 17), and those with unintended hypothermia (*N* = 6). The primary outcome was the Cerebral Performance Categories (CPC) scale at discharge. Of the 48 ECPR patients, 23 patients (47.9%) had good neurological outcomes (CPC 1 and 2) and 27 patients (56.3%) survived to discharge. The normothermia group showed a pattern of higher temperatures compared with the other groups during 48 hours after ECPR. Not only poor neurological outcomes but also intensive care unit (ICU) mortality occurred more often in the unintended hypothermia group than in the other two groups, regardless of the fever control strategy (*p* = 0.023 and *p* = 0.002, respectively). There were no differences in neurological outcomes and ICU mortality between the actively controlled fever group and the normothermia group (*p* = 0.845 and *p* = 0.616, respectively). Unintentionally sustained hypothermia may be associated with poor neurological outcomes after ECPR. These findings suggest that patients who are unable to generate a fever following ECPR may incur severe hypoxic brain injury.

## Introduction

Body temperatures are associated with neurological injuries and other clinical outcomes in comatose patients after return of spontaneous circulation (ROSC) [1–3]. There are many reports regarding therapeutic temperature management and clinical outcomes in patients who have had a cardiac arrest [1, 4–7]. Although there are some debates about the optimal target temperature after cardiac arrest, fever exacerbates acute neurological injury and contributes to poor clinical outcomes after ROSC [1–3, 8–14]. Recently, extracorporeal cardiopulmonary resuscitation (ECPR) has been increasingly utilized to supply oxygenated blood and to provide hemodynamic support in the absence of spontaneous cardiac circulation. Several recent studies have reported that neurological prognoses were better in patients who received ECPR after cardiac arrest than in those who did not receive ECPR after cardiac arrest [15–20]. In patients who had undergone ECPR, extracorporeal circulation and external volume infusion could lower the body temperature [21]. Accordingly, extracorporeal circulation may provide some degree of neuroprotection through induced hypothermia. However, there have been no reports regarding the association between body temperature patterns and neurological outcome in patients receiving ECPR. Therefore, we investigated the association between body temperature patterns and neurological outcomes following ECPR.

## Materials and Methods

### Study Population

This was a retrospective, single-center, observational study of consecutive adult patients who underwent ECPR during hospitalization at the Samsung Medical Center between December 2013 and December 2015. The study was approved by the Institutional Review Board of Samsung Medical Center (SMC 2016-03-035), and we received permission to review and publish information from the patients’ records. Owing to the retrospective nature of the study, the requirement to obtain informed consent was waived.

The initial study group consisted of 72 consecutive patients who underwent ECPR after cardiac arrest and who were unconscious at the time of admission to the hospital (i.e., they had a score <9 on the Glasgow Coma Scale, in which total score ranges from 3 to 15 and lower scores indicate reduced levels of consciousness). From this group, we excluded 24 patients, including those under 18 years of age; those with malignancy whose expected life span was less than 1 year; those whose medical records were insufficient; those who did not survive more than 24 hours after ECPR; and those with a history of head trauma or neurosurgery or a chronic neurological abnormality on admission to the intensive care unit (ICU). Thus, a total of 48 patients who had a witnessed cardiac arrest and were rescued by means of veno-arterial extracorporeal membrane oxygenation (ECMO) support were eligible for this study.

### Definitions and Outcomes

ECPR was defined as both successful veno-arterial ECMO support and “pump on” during cardiac massage in patients with cardiac arrest [17, 22]. In this study, the duration of CPR was defined as the composite time from the onset to the offset of chest compressions. ROSC was defined as the restoration of spontaneous circulation for all rhythms that resulted in more than an occasional gasp, a fleeting palpated pulse, or an arterial waveform. CPR to ECMO pump-on time was defined as the time from the initiation of cardiac massage to activation of the ECMO pump. The primary outcome was neurological status at the time of hospital discharge, according to the Glasgow—Pittsburgh Cerebral Performance Categories (CPC) scale (1 to 5) [23], in which CPC 1 and 2 were classified as good neurological outcomes, while CPC 3, 4, and 5 were considered as poor neurological outcomes. We thoroughly reviewed the medical records, and two independent neurologists determined the CPC scale for each patient.

Body temperature was measured using an ear thermometer and it was controlled with the use of surface cooling devices. If the commercial temperature regulation system involving a hydrogel pad (Arctic Sun, Medivance, Louisville, CO, USA) was not available, we used conventional methods, such as a commercial cooling blanket, with or without the addition of antipyretic agents. In ECPR patients, extracorporeal circulation and external volume infusion could lower their body temperature. Accordingly, extracorporeal circulation may offer some degree of neuroprotection through induced hypothermia. In addition, aggressive therapeutic hypothermia did not performed if the patient had hemodynamic instability and bleeding complications during ECMO. Therefore, in the ECPR setting, surface cooling and the degree of targeted temperature were determined by each intensivist in the ICU according to the SMC therapeutic hypothermia protocol [24]. We classified our patients into the following three groups: actively controlled fever, normothermia, and unintended hypothermia. The actively controlled fever group consisted of patients whose body temperature was controlled by means of surface cooling devices (Arctic Sun or a cooling blanket) with or without antipyretic agents. Surface cooling methods were determined by intensivists in the ICU. Fever was defined as a body temperature above 38°C. Hypothermia was defined as a body temperature below 35°C. The unintended hypothermia group consisted of patients whose mean body temperature was below 36°C until 48 hours in the ICU and who had one or more hypothermic events with no cooling trial on the first day and second day, respectively. Finally, the normothermia group consisted of the remaining patients, whose body temperatures were near 36.5°C and in whom no intentional efforts (e.g., therapeutic hypothermia and warming) had been made to regulate the body temperature.

### Procedure

CPR was performed by the CPR team of the hospital, and all facts related to the CPR scene were recorded by bedside nurses according to the Utstein style guidelines [25]. When CPR was performed for more than 10 minutes or in the event of unstable vital signs or recurrent cardiac arrest, the institutional rapid response team contacted the on-call ECMO team leader, who along with the CPR leader assessed the patient and made a decision about whether to institute ECPR. ECPR was performed when a witnessed cardiac arrest was confirmed, cardiac arrest persisted despite conventional CPR lasting for more than 10 minutes, and the event that caused cardiac was considered reversible [21]. Cases in which ECPR was deferred included cases of short life expectancy (<6 months); cases of terminal malignancy, an unwitnessed collapse, arrest of traumatic origin with uncontrolled bleeding; cases with limited physical activity, an unprotected airway, current massive intracranial hemorrhage, irreversible organ failure or multiple organ failure leading to cardiac arrest; cases in which CPR was undertaken for more than 60 minutes at the time of initial contact; or cases which previously signed “do-not-resuscitate” orders. Age alone did not constitute a contraindication to ECPR [21].

The ECMO team consisted of cardiologists, cardiovascular surgeons, intensivists, special nurses, and perfusionists. Either the Capiox Emergency Bypass System (Terumo, Tokyo, Japan) or the Prolonged Life Support System (Maquet Cardiopulmonary, Hirrlingen, Germany) was used in all cases. A crystalloid solution such as normal saline or balanced solution was used for priming; none of the patients had received blood-primed ECMO. A percutaneous vascular approach was attempted initially in all cases using the Seldinger technique, but when percutaneous cannulation failed, surgical cutdown exposure was performed [21]. The femoral vessels were the most common sites for vascular access, with the use of 14 to 17 French arterial cannulas and 20 to 24 French venous cannulas [22]. Chest compression was stopped once ECMO pump-on was successful during CPR. Anticoagulation was accomplished by a bolus injection of unfractionated heparin, followed by continuous intravenous heparin infusion to maintain the activated clotting time between 150 and 180 seconds. The initial number of revolutions per minute of the ECMO device was adjusted to achieve an ideal cardiac index greater than 2.2 L/min/m^2^ of body surface area, central mixed venous oxygen saturation above 70%, and a mean arterial pressure above 65 mm Hg [22]. Blood pressure was monitored continuously through an arterial catheter, and an artery in the right arm was used for arterial blood gas analysis to estimate cerebral oxygenation. After ECMO, the necessary steps were taken to treat the cause of cardiac arrest, such as percutaneous coronary intervention, coronary artery bypass grafting, heart transplantation, non-coronary cardiopulmonary surgery, and non-cardiopulmonary surgery [22].

### Statistical Analyses

All data are presented as medians and interquartile ranges (IQRs) for continuous variables and as numbers (percentages) for categorical variables. Data were compared using the Kruskal—Wallis test for continuous variables and the chi-square test or Fisher’s exact test for categorical variables. All tests were two-sided, and *P* values <0.05 were considered to indicate statistical significance. Data were analyzed using SPSS statistics version 20 (IBM, Armonk, NY).

## Results

### Baseline and Procedural Characteristics

Of the 48 adult patients with cardiac arrest who underwent ECPR, 25 patients (52.0%) had their body temperature actively controlled by means of surface cooling devices. Arctic Sun was used in 14 patients (29.1%) of this group and a cooling blanket with or without antipyretic drugs was used in 11 patients (22.9%). Seventeen patients (35.4%) who did not undergo intentional regulation of their body temperature (as through therapeutic hypothermia and warming within 48 hours of admission) showed a near-normal temperature pattern, and a slightly elevated body temperature was permitted in these patients. The remaining 6 patients (12.5%) experienced unintended hypothermia, although they did not receive surface cooling devices or antipyretics.

The baseline characteristics of the three groups are presented in Table 1, and the cardiac arrest characteristics are shown in Table 2. There were no significant differences among the three groups except for the initial lactate levels (Tables 1 and 2), with the normothermia group showing lower lactate levels compared with the other two groups. The median age of the study group was 59 years (IQR, 46–70 years), and 35 patients (72.9%) were men.

**Table 1:** Baseline characteristics of the actively controlled fever group, the normothermia group, and the unintended hypothermia group.

Characteristic	Actively controlled fever group (*N* = 25)	Normothermia group (*N* = 17)	Unintended hypothermia group (*N* = 6)	*p*-value
Age (yr)	53 (34–64)	65 (47–70)	65 (60–70)	0.113
Gender, male—no. of patients (%)	18 (72.0)	13 (76.5)	4 (66.7)	0.888
Height (cm)	166 (162–170)	169 (160–170)	166 (160–167)	0.662
Body weight (kg)	65 (58–75)	64 (57–78)	69 (66–74)	0.580
Medical history—no. of patients (%)				
Diabetes mellitus	6 (24.0)	6 (35.3)	4 (66.7)	0.135
Hypertension	8 (32.0)	5 (29.4)	3 (50.0)	0.642
Malignancy	3 (12.0)	2 (11.8)	2 (33.3)	0.380
Dyslipidemia	3 (12.0)	2 (11.8)	0 (0)	0.671
Smoking	12 (48.0)	8 (47.1)	4 (66.7)	0.682
Current smoker	6 (24.0)	5 (29.4)	0 (0)	0.332
Chronic kidney disease	3 (12.0)	3 (17.6)	3 (50.0)	0.100
Previous peripheral arterial occlusive disease	0 (0)	0 (0)	1 (16.7)	0.116
Previous TIA or stroke	1 (4.0)	2 (11.8)	0 (0)	0.473
Previous myocardial infarction	1 (4.0)	3 (17.6)	1 (16.7)	0.316
Previous percutaneous coronary intervention	3 (12.0)	3 (17.6)	1 (16.7)	0.868
Previous coronary artery bypass grafting	0 (0)	1 (5.9)	0 (0)	0.394
Initial Glasgow Coma Scale score—median (IQR)	3 (3–6)	3 (3–3)	3 (3–4)	0.396
Body temperature (°C)—median (IQR)				
First measured body temperature	34.6 (33.2–35.9)	35.0 (34.2–36.0)	35.0 (34.0–35.2)	0.669
Maximum temperature within the first day	36.9 (34.4–37.8)	36.9 (36.5–37.1)	35.9 (35.6–36.5)	0.260
Mean temperature within the first day	35.2 (33.8–37.0)	36.0 (35.7–36.1)	34.7 (34.7–35.5)	0.016
Mean temperature within the second day	35.4 (34.5–37.0)	36.2 (36.1–36.5)	34.9 (34.8–35.4)	0.016
Method of target temperature management—no. of patients (%)				
Arctic Sun	14 (56.0)			
Cooling blanket	11 (44.0)			
Laboratory data on admission—median (IQR)				
Initial lactate (mmol/L)	11.3 (6.1–14.5)	2.7 (2.0–10.0)	9.9 (7.6–11.1)	0.030
Hemoglobin before ECMO (g/dL)	11.9 (9.5–15.2)	10.9 (10.0–12.0)	11.7 (8.9–13.1)	0.458
Hemoglobin after ECMO (g/dL)	11.1 (9.4–12.4)	8.9 (7.7–11.0)	8.7 (7.6–9.7)	0.057
Total bilirubin (mg/dL)	0.8 (0.5–1.0)	0.6 (0.5–1.3)	1.5 (0.8–2.8)	0.479
Blood urea nitrogen (mg/dL)	17.2 (11.9–23.4)	20.8 (15.6–37.8)	23.5 (13.6–24.0)	0.363
Creatinine (mg/dL)	1.3 (1.1–1.7)	1.5 (1.1–1.7)	1.3 (1.0–2.1)	0.945

TIA; transient ischemic attack, IQR; interquartile range, ECMO; extracorporeal membrane oxygenation

**Table 2:** Characteristics of cardiac arrest in the actively controlled fever group, the normothermia group, and the unintended hypothermia group.

Characteristic	Actively controlled fever group (*N* = 25)	Normothermia group (*N* = 17)	Unintended hypothermia group (*N* = 6)	*p*-value
Type of cardiac arrest—no. of patients (%)				0.069
Out-of-hospital cardiac arrest	9 (36.0)	1 (5.9)	1 (16.7)	
In-hospital cardiac arrest	16 (64.0)	16 (94.1)	5 (83.3)	
Bystander-witnessed cardiac arrest—no. of patients (%)	25 (100.0)	17 (100.0)	6 (100.0)	
Bystander CPR—no. of patients (%)	22 (88.0)	17 (100.0)	6 (100.0)	0.229
First monitored rhythm—no. of patients (%)				0.996
Asystole	3 (12.5)	2 (11.8)	1 (16.7)	
Pulseless electrical activity	9 (37.5)	7 (41.2)	2 (33.3)	
Shockable rhythm (VT or VF)	12 (50.0)	8 (47.1)	3 (50.0)	
Defibrillation—no. of patients (%)	17 (68.0)	10 (58.8)	5 (83.3)	0.538
ROSC before ECMO insertion—no. of patients (%)	16 (64.0)	6 (35.3)	3 (50.0)	0.187
CPR duration (min)—median (IQR)	27 (18–45)	27 (18–41)	44 (41–60)	0.085
CPR to ECMO pump-on time (min)—median (IQR)	43(19–52)	30 (18–47)	49 (36–58)	0.247
Location of ECMO insertion—no. of patients (%)				0.075
Intensive care unit	7 (28.0)	7 (41.2)	1 (16.7)	
Cath room	4 (16.0)	6 (35.3)	2 (33.3)	
Emergency room	14 (56.0)	2 (11.8)	2 (33.3)	
Operation room	0 (0)	2 (11.8)	1 (16.7)	
Cardiac cause of arrest—no. of patients (%)				0.181
Acute coronary syndrome	7 (28.0)	9 (52.9)	1 (16.7)	
STEMI	4 (16.0)	5 (29.4)	0 (0)	
NSTEMI	2 (8.0)	4 (23.5)	1 (16.7)	
Unstable angina	1 (4.0)	0 (0)	0 (0)	
Cardiomyopathy	0 (0)	1 (5.9)	2 (33.3)	
Acute aortic syndrome	0 (0)	1 (5.9)	1 (16.7)	
Pulmonary thromboembolism	1 (4.0)	2 (11.8)	0 (0)	
Refractory arrhythmia	7 (28.0)	3 (17.6)	1 (16.7)	
Other	2 (8.0)	2 (11.8)	0 (0)	

CPR; cardiopulmonary resuscitation, VT; ventricular tachycardia, VF; ventricular fibrillation, ECMO; extracorporeal membrane oxygenation, IQR; interquartile range, STEMI; ST-elevation myocardial infarction, NSTEMI; non-ST-elevation myocardial infarction

A cardiac cause was confirmed in 40 patients (83.3%). Acute coronary syndrome was the main cause of cardiac arrest in 17 patients (35.4%), and 8 patients (16.6%) had a history of ischemic heart disease. Refractory arrhythmia was confirmed in 11 patients (22.9%). Cardiac arrest occurred in the hospital in 37 cases (77.0%) and in a different setting in 11 cases (22.9%). The median CPR duration was 32 minutes (IQR, 19–45 minutes), and the median time from CPR to ECMO pump-on was 39 minutes (IQR, 20–54 minutes).

### Neurological and Clinical Outcomes

**Fig 1:**
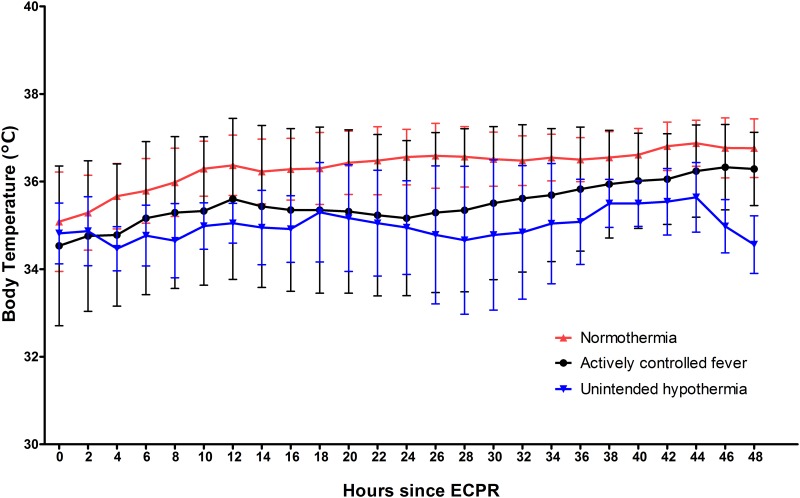
Body temperature patterns in the three patient groups of 48 patients. The temperature curves display the means, and the I bars indicate ± standard deviation. ECPR; extracorporeal cardiopulmonary resuscitation.

**Fig 2:**
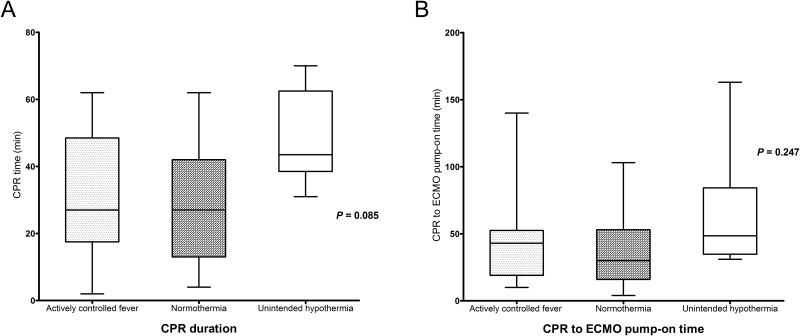
Medians and interquartile ranges for CPR duration (A) and time from CPR to ECMO pump-on (B) for the three body temperature patterns. CPR; cardiopulmonary resuscitation, ECMO; extracorporeal membrane oxygenation.

**Fig 3:**
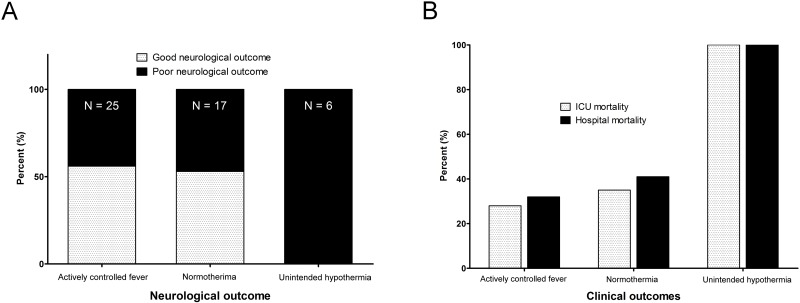
Neurological outcome (A) and clinical outcomes (B) among 48 patients with three body temperature patterns. ICU; intensive care unit.

Of the 48 ECPR patients, successful weaning from ECMO was achieved in 35 patients (72.9%), survival to discharge was noted in 27 patients (56.3%), and 23 (47.9%) patients had good neurological outcomes (CPC 1 and 2). Among the 21 dead patients, 13 patients died of confirmed brain deaths and 8 patients died of multi-organ failure. Although 8 patients died of multi-organ failure, they had possibility of brain death. However, they did not have the confirmatory tests for diagnosis of brain death. All patients of the unintended hypothermia group died of confirmed brain death. During 48 hours after ECPR, body temperature patterns were very similar between the actively controlled fever group (35.4°C; IQR, 34.5–37.0°C) and the unintended hypothermia group (34.9°C; IQR, 34.8–35.4°C); in contrast, the normothermia group showed a pattern of higher temperatures (36.2°C; IQR, 36.1–36.5°C) (Fig 1). Although the three groups did not differ significantly with respect to CPR duration and CPR-to-ECMO pump-on time, these periods were more prolonged in the unintended hypothermia group (Fig 2A and 2B). Not only poor neurological outcomes but also ICU mortality was observed more often in the unintended hypothermia group than in the other two groups, regardless of fever control (*p* = 0.023 and *p* = 0.002, respectively) (Fig 3A and 3B). There were no differences in neurological outcomes and ICU mortality between the actively controlled fever group and the normothermia group (*p* = 0.845 and *p* = 0.616, respectively) (Fig 3A and 3B).

## Discussion

In the present study, we investigated the neurological outcomes and clinical outcomes according to body temperature patterns in patients who underwent ECPR after cardiac arrest. These patterns were very similar between the actively controlled fever group and the unintended hypothermia group, but the normothermia group showed a pattern of higher temperatures compared with the other two groups within the 48-hour period following ECPR. Approximately half of the patients who underwent ECPR had good neurological outcomes. Neurological outcomes and ICU mortality were similar between the actively controlled fever group and the normothermia group; however, unintended hypothermia was associated with a higher incidence of poor neurological outcomes and in-hospital mortality compared with actively controlled fever and normothermia.

To minimize the risk of futile ECMO implantation in the ECPR setting, several methods can be used such as biochemical markers and an end-tidal carbon dioxide (ETCO2) in several previous studies [26–28]. An ETCO_2_ less than 10 mm Hg immediately after intubation and resuscitation more than 20 minutes are associated with extremely poor chances for ROSC and survival [26]. Low lactate clearance, pre-cannulation peripheral venous oxygen saturation ≤8% predicted the development of early multiple organ failure, fibrinogen ≤0.8 g/L and prothrombin index ≤11% were associated with ECPR futility [27, 28]. In our institution, the ECMO team leader and CPR leader determined ECPR futility after discussion among physicians based on reversibility of the cardiac arrest, patients’ comorbidities, life and expectancy as well as laboratory findings.

Thermoregulation is controlled by the central nervous system (CNS). The thermosensitive neurons in the preoptic area of the anterior hypothalamus are considered to be most important for triggering autonomic thermoeffector responses [29, 30]. Thermogenic or thermolytic responses can correct the core temperature toward the set-point level [31], and fever or hypothermia is thought to result from a shift in this set point [32]. The etiology of fever after cardiac arrest may be related to activation of inflammatory cytokines in a pattern similar to that observed in sepsis [33, 34]. Because whole-body ischemia/reperfusion injury triggers a systemic inflammatory response [33], body temperature is commonly elevated after cardiac arrest. Meanwhile, in resuscitated patients, body temperature may decrease after ROSC for a variety of reasons.

Although numerous studies have been conducted to determine the impact of therapeutic hypothermia on a patient’s neurological prognosis, there is limited data regarding the clinical significance of spontaneous or unintended hypothermia in successfully resuscitated patients. Previous studies have suggested that spontaneous hypothermia after cardiac arrest may be due to dysfunction of the CNS thermoregulation center as a result of severe brain damage after prolonged or poorly administered CPR [31, 35, 36]. In addition, a decrease in brain temperature is associated with decreased cerebral blood flow and poor neurological outcomes in patients with traumatic brain injury and intracranial hemorrhage [31, 35, 37]. Similarly, in our study, the patients who had impaired thermoregulation, particularly endothermic heating, had a poor neurological prognosis. However, the relationship between low core temperature and poor neurological outcome needs to be explored further and it should be confirmed in future well-designed studies.

Fever exacerbates acute neurological injury and contributes to poor outcomes after cardiac arrest [1–3, 14]. Although the optimal target body temperature has been the subject of debate, targeted temperature management (TTM) can be used for neuroprotection in eligible adult patients who have been resuscitated from cardiac arrest [7–13]. Several randomized controlled trials demonstrated a decrease in mortality and improvement in neurological outcomes in post-cardiac arrest patients who were treated with TTM [38, 39]. In the present study, neurological outcomes were good in the actively controlled fever group. In the ECPR setting in particular, extracorporeal circulation itself may induce mild hypothermia; therefore, some patients might maintain a normothermic pattern without the need for any cooling devices and may receive some degree of neuroprotection in response to mildly induced hypothermia. Consequently, we suspect that neurological outcomes in the normothermia group may be similar to those in the actively controlled fever group [21].

This study had several limitations. First, it involved a retrospective review of medical records, and the CPC scales were determined on this basis. Although the cause of death had to be accurately verified, its identification was insufficient due to the retrospective nature of this study. Second, the nonrandomized nature of the registry data could have resulted in selection bias. Particularly, the methods used for TTM were determined by each intensivist according to different conditions and situations. Hypothermia can cause coagulopathy; therefore, therapeutic hypothermia was not attempted if patients had substantial bleeding after insertion of the ECMO cannula. Therefore, we used surface cooling devices such as Arctic Sun in a limited number of the patients. Third, body temperature was measured using tympanic temperatures in all cases, but core temperature was measured in only a limited number of patients. Fourth, there were a limited number of subjects in each subgroup, particularly, in the unintended hypothermia group. Nevertheless, the cause of death was brain death in all patients of unintended hypothermia group. The number of enrolled patients is not sufficient to infer definite conclusions from this study, but it may help in generating hypotheses on which further research may be based. Lastly, since we excluded 7 patients who did not survive for more than 24 hours after ECMO initiation in final analysis, the survival rate in this study seem to be better than those of previous studies. Actual survival rate in all ECPR patients during the same period is similar to previous studies [22, 40, 41]. Our study was conducted in a small cohort at a single institution. Therefore, future studies with larger cohorts are needed to confirm our findings.

## Conclusions

In the ECPR setting, neurological outcomes and mortality were similar between the actively controlled fever group and the normothermia group, but unintentionally sustaining hypothermia may be associated with poor neurological outcomes. These findings suggest that patients who are unable to generate a fever following ECPR may incur severe hypoxic brain injury.
